# Supplementary material for: Grasping through dynamic weaving with entangled closed loops
Source: Nat Commun. 2023 Aug 2;14:4633. doi: 10.1038/s41467-023-40358-y (PMC10397280; doi:10.1038/s41467-023-40358-y)
Supplement: Supplementary file 1 — Supplementary Information [file 41467_2023_40358_MOESM1_ESM.docx]

**
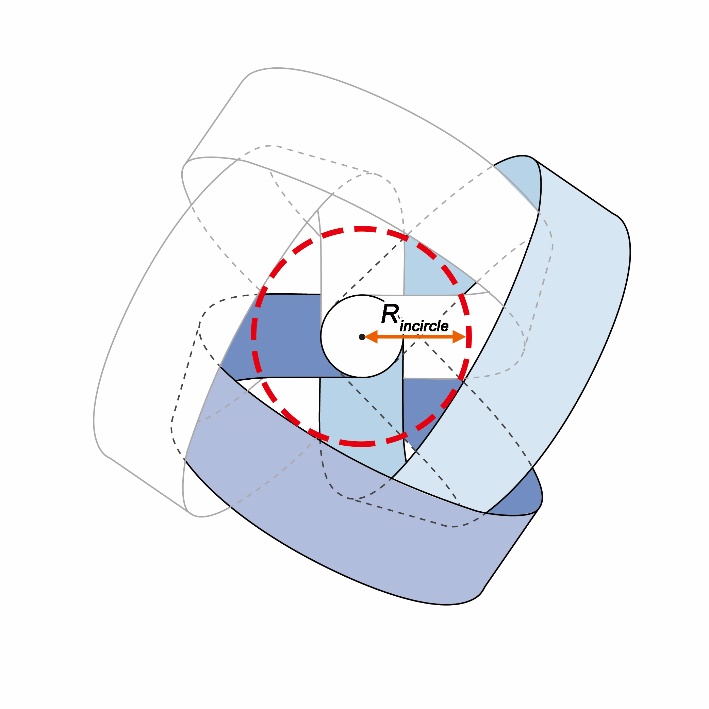
**

**Supplementary Fig. 1** | The completeness of weaving is quantified through an imaginary inscribed circle with radius $R_{\mathrm{incircle}}$, where the inner edges of the strips were tangents in weaving gripper.

**
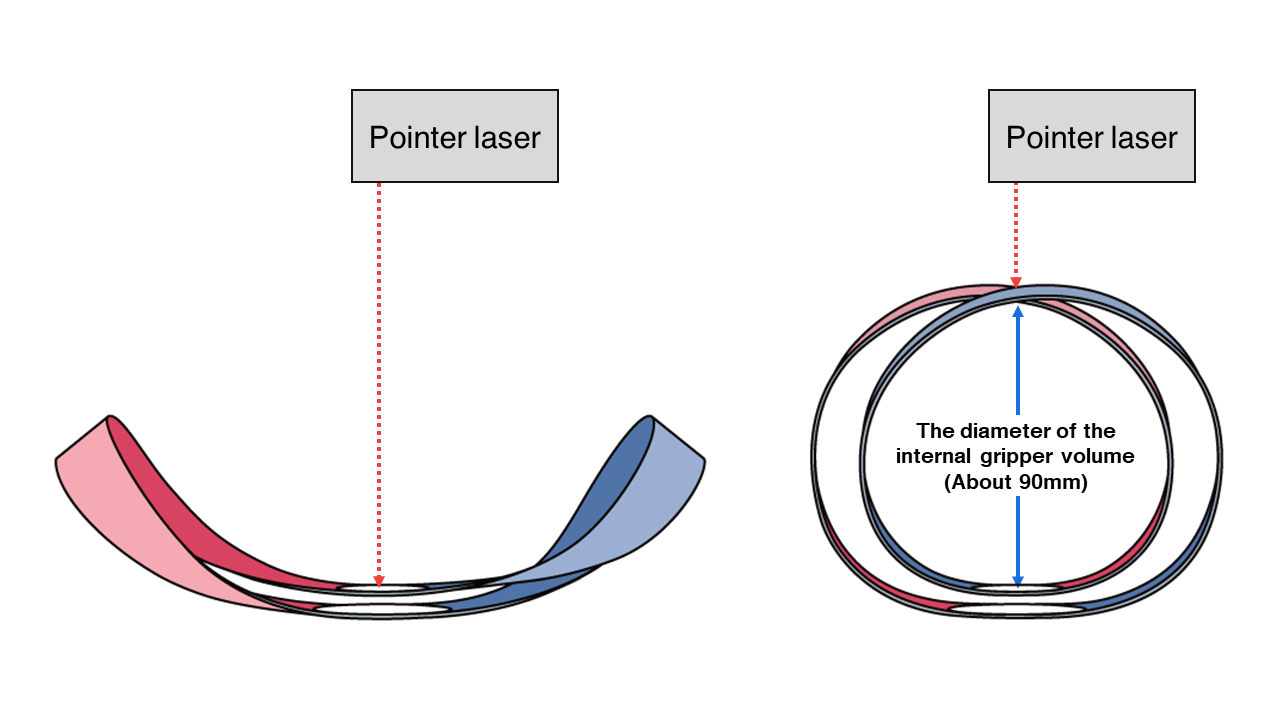
**

**Supplementary Fig. 2** | The internal volume of the gripper: We assumed that the gripper has an ideal spherical shape. To determine the diameter of the gripper, we measured the distance from the top to the bottom of the closed gripper using a pointer laser sensor (IL-600, Keyence), which resulted in an approximately 90 mm. The diameter of the 90 mm ball is 100% object size compared to the internal gripper volume, assuming that the diameter of the 90 mm sphere is similar to the internal volume of the gripper.


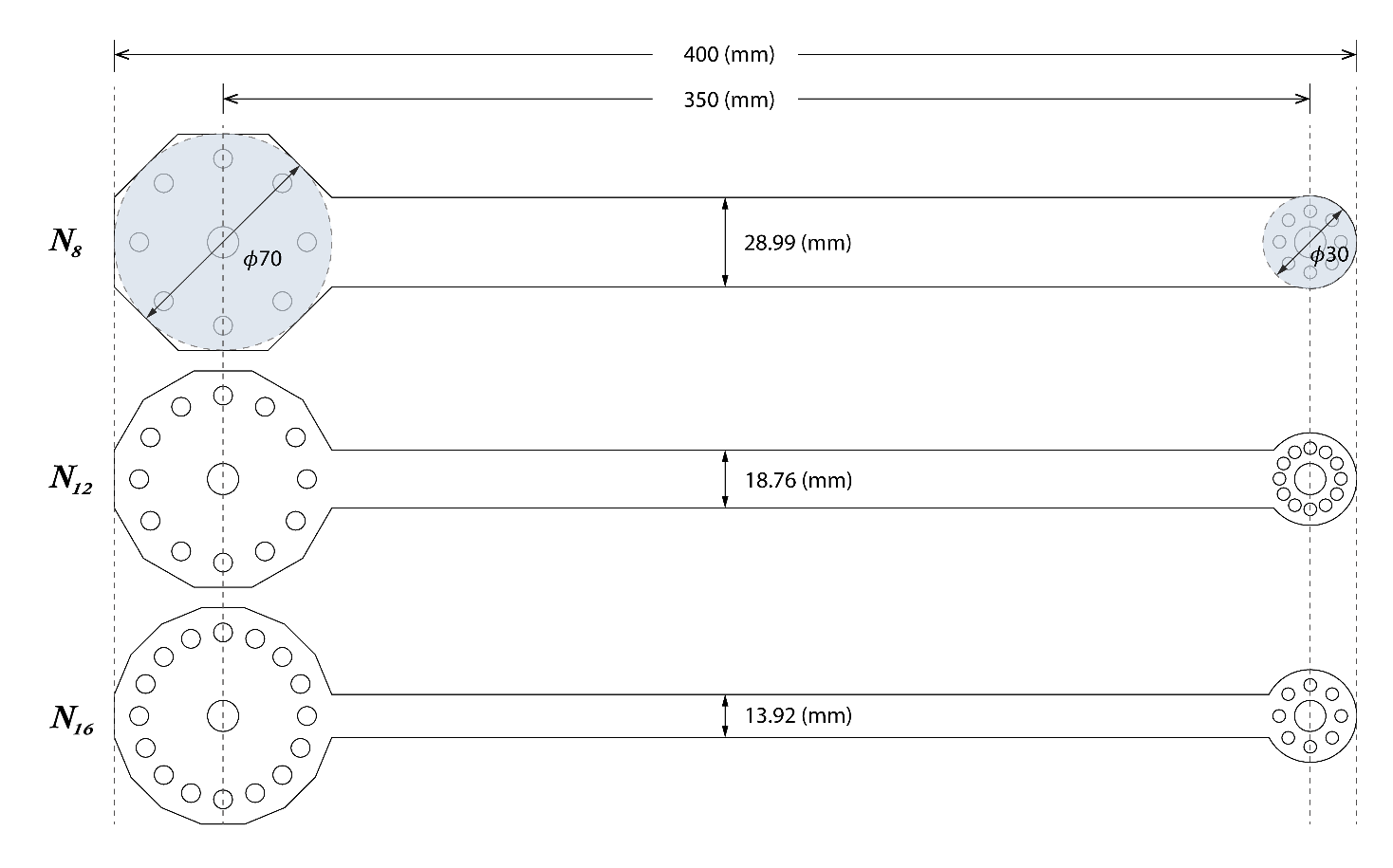
 **Supplementary Fig. 3** | The weaving grippers classified by *N* (considered in Fig. 3 of the main text): $N_{8}, N_{12},N_{16}$. Each strip is designed in a linear shape with plates at both ends. The outer plate is a polygon with a 70mm diameter, and the inner plate is circular with a 30mm diameter. The total strip length to outer plate radius ratio is 10:1. All the design parameters of the grippers ($N_{8}$, $N_{12}$, and $N_{16}$) were kept the same, and only changed the outer plate by configuring the polygon within the same outer circle. By adjusting the width of the strip based on the polygon with *N* sides in the same outer circle, increasing *N* brings the total area closer to the surface of a sphere.


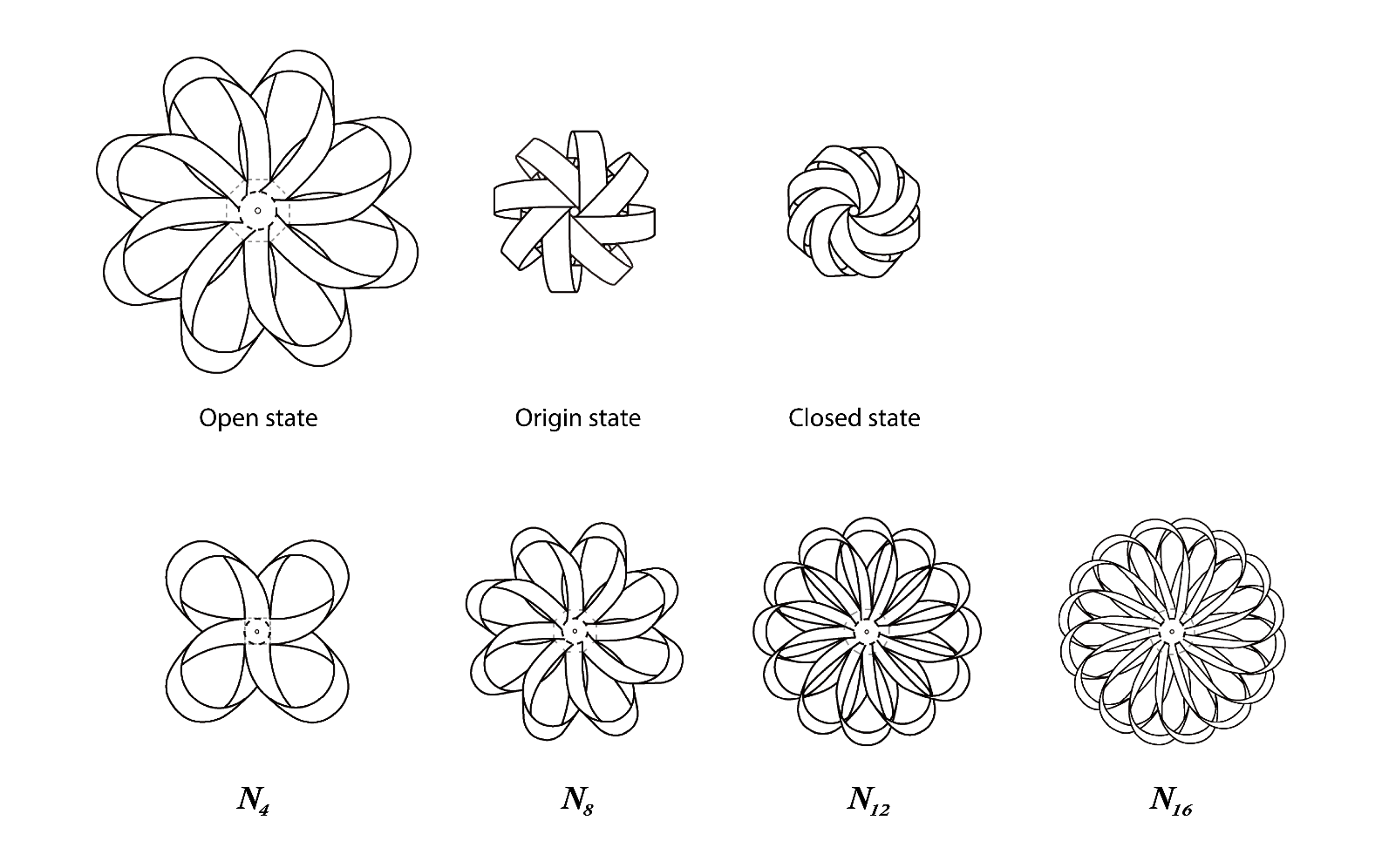


**Supplementary Fig. 4** | Three states of the weaving gripper: Open state, Origin state, and Closed state. The loops change shape through relative rotation, with shape dependent on *N*.


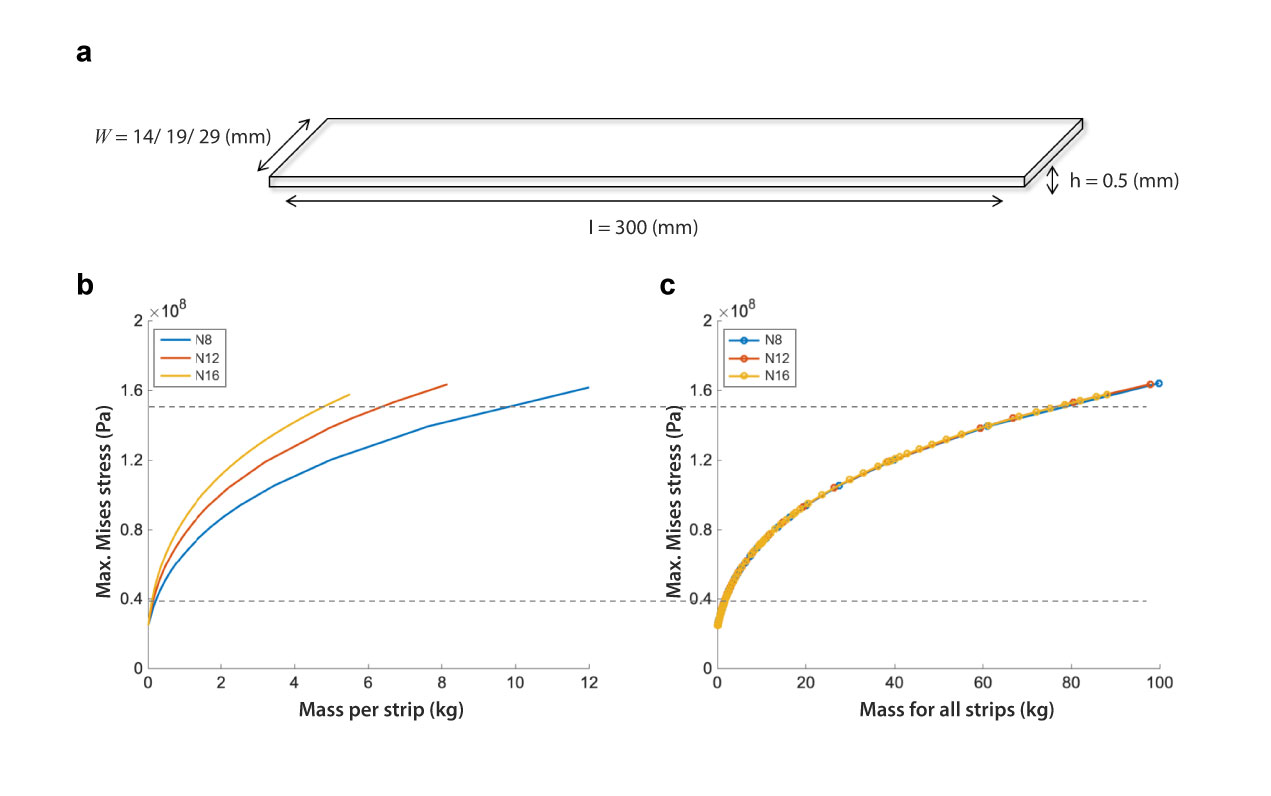
 **Supplementary Fig. 5** | **a** A scheme for the toy model for the circularization (*W* ≅ 14 mm at $N_{16}$, 19 mm at $N_{12}$, 29 mm at $N_{8}$). Numerical simulations using a commercial FEA software package, ABAQUS (Standard): **b** The maximum Mises stress of the strip and the load applied by the indentation to the strip. A single bent PET strip fixed at both ends was indented by imposing a 90mm diameter rigid sphere in a vertical position at the center of the strip. **c** The calculated load that the gripper experienced by multiplying the load applied to a single strip with total number of strips. Assuming the PET film has a yield strength of 150 MPa, the gripper could withstand a load of up to 75 kg regardless of the number of strips.


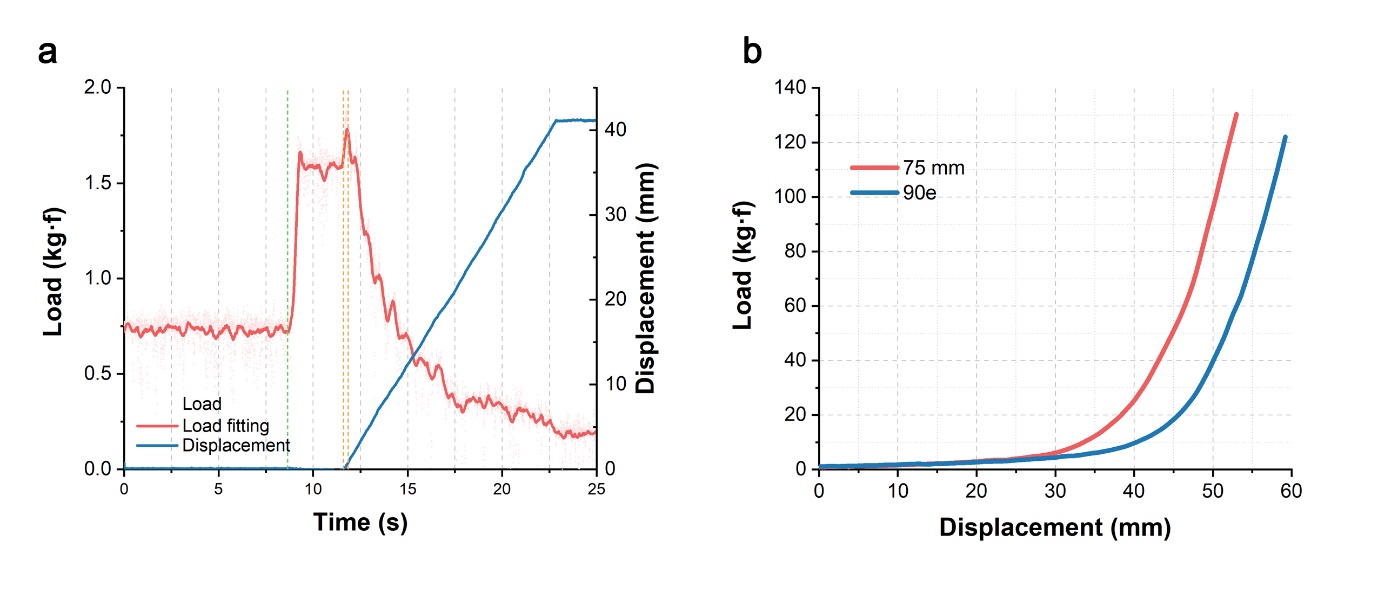
 **Supplementary Fig. 6** | **a** The representative data of load capacity and displacement over time with $B_{105}$. The green line denotes the moment when the gripper contacts the surface of$B_{105}$, transitioning from open state ($\theta_{i}=-110^{\circ}$) to $\theta_{a}=20^{\circ}$. The orange line is the moment when the gripper starts to be pulled up, resulting in slipping on the surface of $B_{105}$. **b** The representative data of load capacity data according to the displacement using $B_{75}$ and an ellipsoid ball $B_{90e0.9}$.


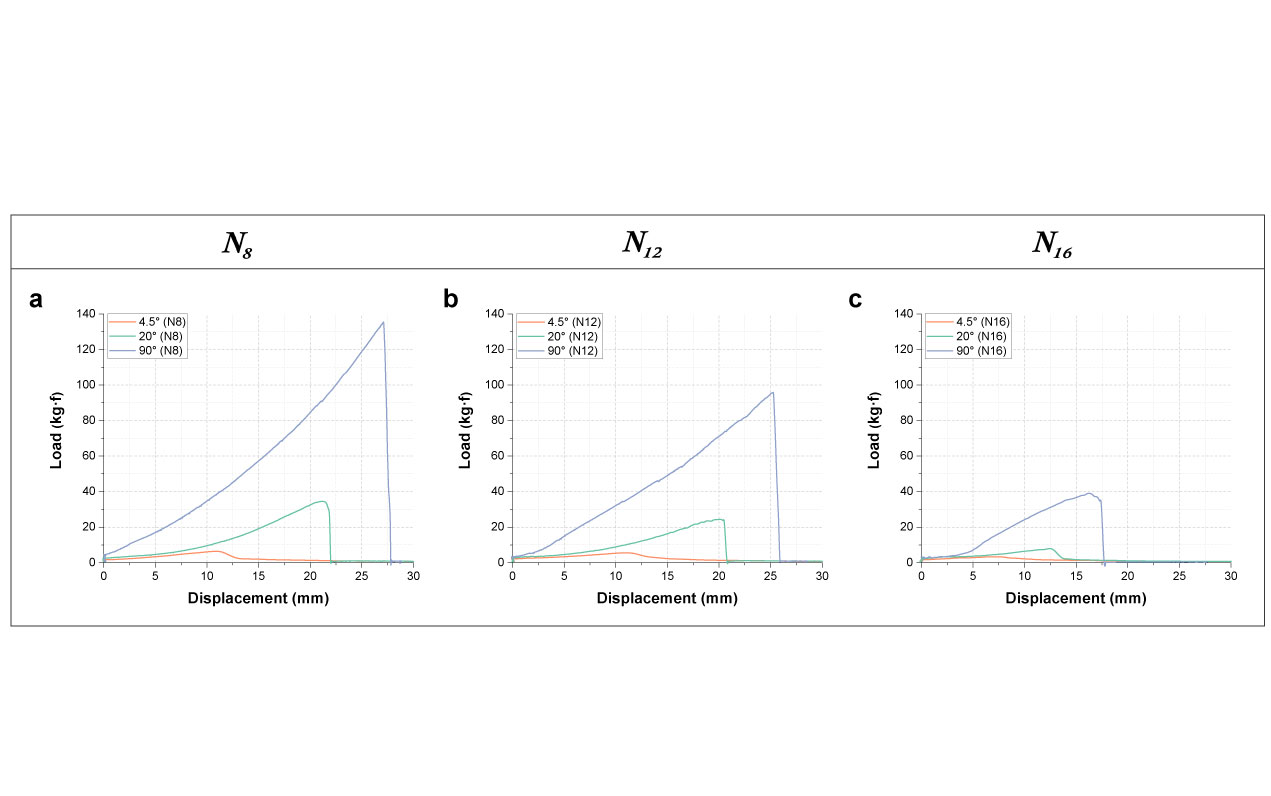
 **Supplementary Fig. 7 |** The experimental data of load capacity for $N\left( \mathbf{a} N_{8}, {\mathbf{b} N}_{12}, \mathbf{c} N_{16} \right)$ using $B_{90}$.


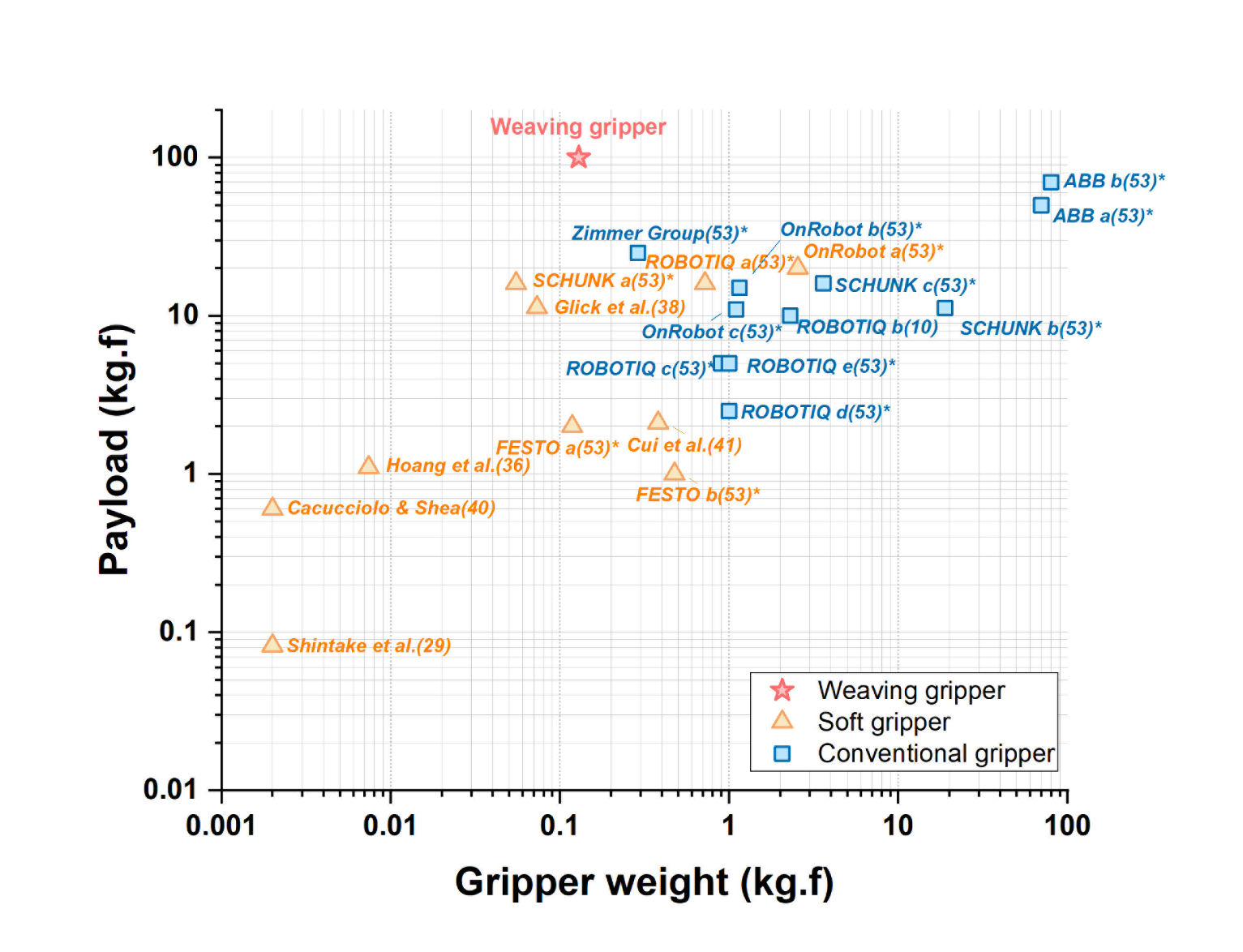


*Some of the data referenced in the brochure is included.

**Supplementary Fig. 8 |** The comparison of gripper weight to payload capacity for a weaving gripper (red), soft grippers (yellow), and conventional grippers (blue).
