# Supplementary material for: Grasping through dynamic weaving with entangled closed loops
Source: Nat Commun. 2023 Aug 2;14:4633. doi: 10.1038/s41467-023-40358-y (PMC10397280; doi:10.1038/s41467-023-40358-y)
Supplement: Supplementary file 3 — Description of Additional Supplementary Files Document [file 41467_2023_40358_MOESM3_ESM.pdf]

Description of Additional Supplementary Files Document

Supplementary Movie 1 - Gripper operation video considered in Fig. 2.

Supplementary Movie 2 - Gripper load capacity evaluation: grasping three 30 kg·f dumbbells (90 kg·f), and a 68 kg·f man.

Supplementary Movie 3 - Gripper adaptability evaluation: grasping a wood block (60x60x60 mm), four golf balls (Dia.: ~43 mm), a playing card (Thickness: ~0.15 mm), a M3 nut, three rubber ducks (~30 mm) on the water, and a flower with a medium scale gripper (G_m) considered in Fig. 4.

Supplementary Movie 4 - Gripper size variation: grasping 1 cent coin, M6 nuts, 7 mm four beads, toys, and a 0.5 kg·f weight with a small size gripper (G_s) & grasping a paper box with 5 kg·f of flour, 6 packs of 2L bottled water (12 kg·f), and a 60 kg·f dumbbell with a large size gripper (G_l) considered in Fig. 4.
